# Ventriculoperitoneal Shunt Surgery for Hydrocephalus: One of the Common Neurosurgical Procedures and Its Related Problems

**DOI:** 10.7759/cureus.35002

**Published:** 2023-02-15

**Authors:** Farrukh Javeed, Anmol Mohan, Um Ul Wara, Lal Rehman, Maham Khan

**Affiliations:** 1 Neurological Surgery, Jinnah Postgraduate Medical Centre, Karachi, PAK; 2 Medicine, Karachi Medical and Dental College, Karachi, PAK; 3 Medicine and Surgery, Karachi Medical and Dental College, Karachi, PAK; 4 Neurosurgery, Jinnah Postgraduate Medical Centre, Karachi, PAK; 5 Radiation Oncology, Aga Khan University Hospital, Karachi, PAK

**Keywords:** shunt failure, cerebrospinal fluid (csf), shunt complications, ventriculoperitoneal shunt, hydrocephalus

## Abstract

Objective

This study was conducted to assess the outcome in patients with hydrocephalus who underwent ventriculoperitoneal (VP) shunt surgery.

Methods

This retrospective study was conducted at the neurosurgery department of a tertiary care hospital. The time frame was three years and five months from January 2017 to May 2020 with a follow-up of six months.

Results

This study included 1030 patients, out of whom 64.2% were male and 35.8% were female. While the majority of the patients were more than 11 years of age (466), age did not have any significant impact on the outcome of the ventriculoperitoneal shunt surgery. The most common cause of hydrocephalus was congenital (47.6%). A good outcome was seen in 63.4%, mortality was 10.6%, and complications were identified for 25.8%. The underlying pathology had a significant impact on the outcome in our study (p-value < 0.05) where the congenital cause of hydrocephalus showed a better outcome than any other cause.

Conclusion

Ventriculoperitoneal shunt is a good way to manage hydrocephalus, but there is always a high risk of complications.

## Introduction

Hydrocephalus is a condition characterized by the buildup of excess cerebrospinal fluid (CSF) in the ventricles of the brain resulting in active distension of the ventricular system. The frequency globally is estimated at about 0.9-1.2/1000 [1]. It occurs because of increased CSF production, decreased reabsorption via arachnoid granulations, or blockage of normal flow of CSF that is frequently seen in tumors, myelomeningoceles, arachnoid cysts, etc., which ultimately raises the intracranial pressure (ICP) [2].

The gold standard and most common neurosurgical procedure used to manage hydrocephalus is ventriculoperitoneal (VP) shunt surgery, while the other surgical alternatives include endoscopic third ventriculostomy (ETV), ventriculopleural (VPL) shunt, and ventriculoatrial (VA) shunt [2,3]. Ventriculoperitoneal shunt surgery involves the placement of a VP shunt, which is a medical device that drains the excess cerebrospinal fluid into the peritoneal cavity where the fluid is absorbed.

There are mainly two types of VP shunts based on the type of valve used: programmable (adjustable differential pressure valves) and non-programmable (fixed differential pressure valves). A programmable shunt utilizes an adjustable pressure setting valve that regulates the flow of CSF from outside the body without another surgical procedure, while a non-programmable shunt has a prefixed pressure setting [2,4]. A study published in 2017 found that although programmable shunts are more expensive, they have fewer chances of having any complications. Therefore, the difference in pricing is greatly reduced when the cost of complication surgeries is considered [4].

Despite being a well-established procedure in modern medical practice, there is always a possibility for shunt malfunction and failure with a rate of about 40%-50% for the pediatric population and about 30% for adults [5]. Only about 30%-37% of VP shunts remain successful and do not require revision and replacement during the first 10 years of shunt placement [6]. In the first year after shunt installation, however, the incidence of VP shunt failure has been reported to be 11%-25% [7]. Shunt failure is a sequel of multiple complications, which include obstruction (either complete or partial), shunt infection (most common being *Staphylococcus epidermidis*, which is a part of skin flora), overdrainage leading to subdural hematoma (SDH), underdrainage, shunt migration, peritoneal pseudocysts, bowel perforation, and hernias [1,6-8]. Shunt malfunction remains the most frequent reason for shunt revision and replacement often requiring frequent and long hospital stays. However, with proper patient selection and adequate intraoperative measures, the frequency of shunt failure can be reduced evidently [9].

## Materials and methods

Study setting

Our study was conducted at the department of neurosurgery at Jinnah Postgraduate Medical Centre (JPMC), Karachi.

Study type and duration

This study was a retrospective observational study where data was taken from January 2017 to May 2020.

Inclusion/exclusion criteria

We included patients of all ages and both genders who underwent VP shunt procedure for hydrocephalus (diagnosed clinically and radiologically) due to any pathology. Patients with a previous history of VP shunt surgery were excluded.

Data collections

The data was collected, after Institutional Review Board approval, from the record and documents of each patient where the clinical history, examination, diagnosis, and preoperative and postoperative computed tomography (CT) scans and follow-up notes were assessed. Only patients with complete preoperative and postoperative information were included, and their findings were collected on a standardized data collection form.

Assessment

The frontal/occipital horn ratio (FOR) was used to assess the severity of hydrocephalus: mild (0.36-0.41), moderate (0.42-0.54), and severe (≥0.55). All surgical procedures were done by a neurosurgeon with at least two-year experience in neurosurgery, under general anesthesia. Ventricular catheter was passed through Keen's point in all patients. The tip of the catheter in the ipsilateral frontal horn was deemed the optimal location for the shunt on the basis of the postoperative CT scan.

Data analysis

Frequency and percentages were calculated for qualitative variables such as gender, underlying pathology, type of procedure, type of shunt used, and outcome. Data was stratified to deal with confounding factors such as age, gender, and underlying pathology. The outcome was defined as good outcome (where patients improved without any complication or mortality) and bad outcome (where patients died or encountered any complication(s) of VP shunt). Chi-square test was applied, and p-value ≤ 0.05 was taken as significant.

## Results

Gender

A total of 1030 patients were included in our study who underwent VP shunt, of which 662 (64.2%) were male while 368 (35.8%) were female patients.

Age

Patients of more than 11 years of age were the most common group with 466 (45.2%) patients, while patients of age 1-11 were 310 (30.1%) and less than one year were 254 (24.7%).

Underlying pathology

The most common cause of hydrocephalus in our study was congenital with 490 (47.6%) patients, out of which 355 (72.4%) had obstructive hydrocephalus. Other pathologies were neoplastic lesions in 354 (34.4%), meningitis in 148 (14.3%), and miscellaneous in 38 (3.7%).

Radiological assessment of hydrocephalus

Preoperative evaluation of CT scan showed that patients who had moderate hydrocephalus were 488 (47.3%) and severe hydrocephalus were 542 (52.6%).

Type of procedure

From a total of 1030 surgeries, 747 (72.5%) were done as elective procedures, while 283 (27.4%) were emergency procedures.

Shunt types

All patients underwent non-programmable medium pressure shunt placement. Chhabra (Surgiwear, Shahjahanpur, India) was the most frequently used shunt among our patients who underwent VP shunt surgery, accounting for 894 (86.7%) patients, while few other types of shunts were also used as mentioned in Table 1. 

**Table 1:** Types of shunts used.

Shunt types	Number (n)	Percentage (%)
Chhabra	894	86.7
Integra	56	5.4
Medtronic	37	3.5
Bhatti	28	2.7
BMI	15	1.4

Outcome

Postoperative radiological assessment showed that after VP shunt placement, 891 (86.5%) patients had no hydrocephalus, and 139 (13.4%) patients had only mild hydrocephalus. Clinically, good outcome was observed in 654 (63.4%) patients and bad outcome in 376 (36.5%). A bad outcome was seen due to mortality and complications. The overall mortality on six-month follow-up was 10.6% (110). In our study, the age of the patient did not have a significant impact on the outcome of VP shunt procedure (p-value > 0.05), but the type of procedure (elective versus emergency) showed a significant relation with the outcome. The underlying pathology also showed a significant role in determining the outcome of VP shunt (p-value < 0.05) (Table 2).

**Table 2:** Outcome of ventriculoperitoneal shunt procedure according to underlying pathology.

Underlying pathology	Good outcome	Bad outcome	Total	P-value
Congenital	318	172	490	<0.05
Neoplastic	206	148	354	
Meningitis	103	45	148	
Miscellaneous	27	11	38	
Total	654	376	1030	

Complications

The complication rate was 25.8% (266 patients) in our study group (Table 3) with shunt malfunction being the most common one in 111 patients.

**Table 3:** Number of complications encountered after ventriculoperitoneal shunt.

Complications	Number (n)	Percentage (%)
Shunt malfunction	111	10.7
Infections	99	9.6
Abdominal complications	40	3.8
Overdrainage	16	1.5

## Discussion

The management of hydrocephalus has been a challenging task for every neurosurgeon worldwide because of the complex dynamics of cerebrospinal fluid (CSF) and its effects on the brain. Ventriculoperitoneal shunt operations have remained a prominent and reliable technique for the treatment of hydrocephalus but are nevertheless at risk for a large variety of complications and revisions [2].

VP shunt surgery requires keen observation and care after its placement in order to prevent shunt failure and a requirement of its revision that puts a lot of financial pressure on the patients; therefore, a suitable model of shunt system is used. Shunt systems come in a variety of different configurations and models including Medtronic (Medtronic Minimally Invasive Therapies, Minneapolis, MN, USA), ​Integra (Integra NeuroSciences, Princeton, NJ, USA), BMI (Wellong Instruments, Taipei City, Taiwan), Chhabra (Surgiwear, Shahjahanpur, India), and Bhatti (Pakistan). The Chhabra shunt has numerous benefits and is less expensive as it is a commonly used shunt system in Pakistan. It is used in more than 50 countries worldwide [2,10,11]. Its catheters are made of barium sulfate, which makes it easily detectable on an X-ray. In our study, the majority of the patients with hydrocephalus were implanted with a Chhabra shunt because of its non-necrotic properties on the skin, as well as a flow-regulated valve it contains maintaining the opening pressure [10].

With more than a thousand patients in our research, a peculiar observation was made that the number of male patients was significantly higher than the female patients with a male-to-female ratio of 2:1, which is consistent with many previous reports [12-14]. ​It might be a consequence of the traditional society's male-dominated structure, social traditions, and cultural trend toward the availability of more treatment facilities to males than females [12].

A considerable improvement was observed on the basis of change in pre-op and post-op Evans ratio in our patients with a p-value < 0.05. The majority of patients had no hydrocephalus after the VP shunt placement, and only 13.4% of patients had mild hydrocephalus. In our literature review, we found similar results with only 16% of patients having post-op mild hydrocephalus, and the remaining had no hydrocephalus [15]. A clinical good prognosis was observed in two-thirds of our patients, while one-third of patients had a bad prognosis. The bad prognosis was the result of mortality rate and complications. The mortality rate of the patients that underwent VP shunt ranged from 5% to 15% in the literature review [1,16,17]. Our study evaluated an overall mortality rate of ​10.6% on six​-month follow-up.

Despite using a highly effective shunt system and keeping patients under strict observation, there is always a possibility for shunt failure because of either shunt malfunction, infection, abdominal manifestations, or overdrainage, which can result in increased morbidity and mortality of the patients [8,17,18]. Therefore, the patients that undergo VP shunt surgery must be assessed for any postoperative complications at regular intervals. The VP shunt failure rate reported in previous studies frequently ranged 18% to 28% or even higher in some [17-21]. In our retrospective study, we found out that the contemporary rate for VP shunt complications was 25.8%. The most common complications in our study were shunt malfunction and shunt infections, both accounting for more than three-quarters of total patients.

Our demographic data included members from various age groups, most of whom were patients over ​11 ​years of age, accounting for approximately half of total patients​, making this age group the most common among others, while one-fifth of patients were less than one year of age. The ratio of good outcome to bad outcome was similar in all age groups showing that the age of the patient did not have any significant impact on the VP shunt procedure.

Congenital anomalies were the most common associated diagnosis, which was present in almost half of the patients. Other common underlying pathologies were brain tumor (34%) and meningitis (15%). More than two-thirds of patients that had meningitis had a good outcome, while only less than two-thirds of patients with congenital anomalies and neoplasia had a good outcome. The findings of our research thus indicate improved results for the patients with meningitis.

The ratio between elective shunt procedure and emergency procedure was 3:1. More than two-thirds of the patients who had an elective procedure had a good outcome, while more than half of the patients who underwent emergency surgery had a bad outcome. So, these results show a strong and significant relationship between the type of procedure performed and the outcome. The likelihood of a good outcome is much greater in patients who are subjected to an elective operation compared to emergency patients.

Based on the retrospective design of our study, our data has some demographic and analytical constraints and limitations because only those patients' data has been taken, which proved to be accurate and reliable, and moreover, patients with lost and incomplete data were omitted from participation. Despite these precursive drawbacks, this study contributes greatly to the science reservoir of knowledge and information.

## Conclusions

Ventriculoperitoneal shunt is the most frequent procedure used to treat hydrocephalus, but despite the wide experience of operating neurosurgeons, the complication rate is still high. We conclude that the findings in our study have been influenced considerably by the underlying pathology and the type of procedure in VP shunt patients. Younger patients with congenital hydrocephalus had better prognosis when operated with shunt placement in an elective setting.

We suggest that proper patient selection, the use of sterile surgical techniques and equipment, improving the quality of VP shunt devices, and definite treatment of the primary pathology causing hydrocephalus are the important factors that must be taken into account to benefit the good outcome of the VP shunt placement.
